# LncRNA GACAT1 targeting miRNA-149 regulates the molecular mechanism of proliferation, apoptosis and autophagy of oral squamous cell carcinoma cells

**DOI:** 10.18632/aging.203416

**Published:** 2021-08-30

**Authors:** Jingxin Chen, Xubin Chen, Liangbin Fu, Jimin Chen, Yang Chen, Feng Liu

**Affiliations:** 1 Department of Oral and Maxillofacial Surgery, Hainan Province People’s Hospital, Haikou, Hainan, China; 2 Department of Pathology, Hainan Province People’s Hospital, Haikou, Hainan, China; 3 Department of Stomatology, Hunan Province People’s Hospital, Changsha, Hunan, China

**Keywords:** lncRNA GACAT1, miRNA-149, oral squamous cell carcinoma, proliferation

## Abstract

To explore the effects of lncRNA GACAT1/miR-149 molecular axis on the proliferation, apoptosis, migration and autophagy of oral squamous cell carcinoma (OSCC) cells, and to explore its molecular mechanism. The expressions of lncRNA GACAT1 and miR-149 in tissues and cell lines of patients with OSCC were detected by qRT-PCR. Si-control, GACAT1-siRNA, inhibitor NC and miR-149 inhibitors were transfected into OSCC cells separately or in combination with Lipofectamine 2000. The binding sites between lncRNA GACAT1 and miR-149 were predicted using the miRanda website, and the targeting relationship was verified by dual-luciferase assay. The expression of lncRNA XIST and miR-149 was detected by qRT-PCR. CCK-8 assay was used to detect cell activity. Cell cycle distribution and apoptosis were detected by flow cytometry. Cell migration ability was detected by Transwell assay. The expression of migration and autophagy-related proteins was detected by western blot. LncRNA GACAT1 was highly expressed in cancer tissues and cell lines of OSCC patients (*P* < 0.01), while miR-149 was low expressed (*P* < 0.01). LncRNA GACAT1 binds to miR-149 targeting. The down-regulation of lncRNA GACAT1 inhibited the proliferation and migration of OSCC cells and promoted apoptosis and autophagy (*P* < 0.01). The transfection of miR-149 inhibitor had the opposite effect. Knockdown of lncRNA GACAT1 and transfection with miR-149 inhibitor reversed the effect of GACAT1 silencing on OSCC cells. Inhibition of lncRNA GACAT1 can inhibit the proliferation and migration of OSCC cells, promote apoptosis and autophagy, and the mechanism may be related to the targeting of miR-149.

## INTRODUCTION

Oral squamous cell carcinoma (OSCC) is one of the most common malignant tumors in the head and neck. It has the characteristics of strong local invasion, cervical lymph node metastasis and high recurrence rate [1, 2]. At present, there is no effective tumor marker for the clinical diagnosis and treatment of OSCC. Therefore, exploring the key factors in the occurrence and development of OSCC and elucidating its mechanism will help to find new markers and therapeutic targets for the diagnosis of OSCC.

With the progress and development of biotechnology such as genome sequencing, it has been confirmed that more than 97% of the transcripts in the human genome do not have the function of coding protein, that is, non-coding RNA (ncRNA), which mainly includes small non-coding RNA (miRNA) and long non-coding RNA(lncRNA) [3]. MiRNAs generally have 18 to 25 nucleotides, and their sequences are highly conserved in evolution. They regulate gene expression by targeting specific mRNA. It plays an important role in cell proliferation, apoptosis, differentiation and metabolism. Studies have shown that miR-149 is involved in the occurrence and development of tumor through a variety of mechanisms. Its low expression in prostate cancer, breast cancer and other tumors affects the growth, invasion, migration and other biological processes of cancer cells [4, 5]. Song et al. showed that miR-149 was low expressed in OSCC cells, but its molecular mechanism was not clear [6]. Therefore, it is necessary to further explore the regulatory mechanism of miR-149 in order to provide a new therapeutic target for the biological treatment of OSCC.

One of the modes of interaction between lncRNA and miRNA in tumor regulation is to act as endogenous competing RNA of miRNA (ceRNA), inhibit the expression of miRNA, and then affect the regulation of downstream genes of miRNA [7, 8]. For example, lncRNA MIAT can bind miR-1301-3p through “sponge” effect, and then form lncRNA MIAT/miR-1301-3p/INCENP regulatory pathway with the target gene INCENP of miR-1301-3p, which affects the progress of esophageal squamous cell carcinoma [9]. A large number of evidences indicate that lncRNA GACAT1 is involved in the occurrence and development of tumors [10–12]. It is overexpressed in many tumor cells, and inhibition of lncRNA GACAT1 expression can not only terminate cell transformation, but also reverse cell malignant transformation. But so far, there are no reports about lncRNA GACAT1 and OSCC. In this study, we used biological methods and online database miRanda to predict and found that there were possible regulatory targets between miR-149 and lncRNA GACAT1. Based on this, we speculate that lncRNA GACAT1 may participate in the occurrence and development of OSCC by down regulating the expression of miR-149.

## METHODS

### Human samples

Twenty cases of OSCC were collected from January 2017 to January 2020 and resected in Hainan Provincial People’s Hospital. All patients were primary OSCC, and all patients had not received radiotherapy, chemotherapy and other related tumor therapy. In addition, the adjacent tissue (more than 5cm away from the cancer tissue) was selected as the control group. All patients signed informed consent. This study was approved by the Ethics Committee of Hainan Provincial People’s Hospital. The collected samples were immediately frozen in liquid nitrogen for 5 minutes and then stored for a long time at −80°C. The clinicopathological characteristics of patients were summarized in Table 1.

**Table 1:** The expression levels of lncRNA GACAT1 and miR-149 in OSCC patients with different clinicopathological features.

**Characteristics**	**Case, *n* = 20**	**GACAT1 level**	***P***	**miR-149 level**	***p***
Age (years)			0.418		0.623
<60	8	2.91 ± 0.64		0.52 ± 0.13	
≥60	12	2.79 ± 0.73		0.57 ± 0.17	
Sex			0.113		0.541
Female	9	3.01 ± 1.02		0.61 ± 0.21	
Male	11	2.66 ± 0.69		0.49 ± 0.16	
TNM stage			0.016		0.024
I-II	8	2.33 ± 0.58		0.77 ± 0.29	
III-IV	12	3.26 ± 0.92		0.40 ± 0.11	
Local infiltration			0.618		
Yes	6	2.86 ± 0.72		0.56 ± 0.18	
No	14	2.74 ± 0.86		0.54 ± 0.14	
Lymph node metastasis			0.009		0.003
Yes	7	3.31 ± 1.09		0.38 ± 0.19	
No	13	2.39 ± 0.84		0.82 ± 0.33	

### Cell culture and transfection

OSCC cell lines (PECAPJ41 and HSC-4) and normal oral cells (HOK) were purchased from Nanjing Kebai Biotechnology Co., Ltd (Nanjing, China). PECAPJ41 and HSC-4 cells were cultured with RPMI 1640 (Thermo Fisher Scientific, USA) containing 10% fetal bovine serum (FBS). HOK was cultured in MEM (Thermo Fisher Scientific, USA) containing 10% FBS. All cells were cultured in a constant temperature and humidity box at 37°C and 5% CO_2_. siRNA negative control (si-control), GACAT1-siRNA, inhibitor NC and miR-149 inhibitors were constructed by Geneseed Co., Ltd. (Guangzhou, China). Cells were cultured in 96 well plates 24 hours before transfection. According to the manufacturer’s protocol, Lipofectamine 2000 (Invitrogen, CA) was used for transfection.

### Cell proliferation was detected by cell counting Kit-8 (CCK-8)

Cell viability was measured by CCK-8 (Dojindo, Japan). The transfected cells were seeded into 96 well plates. Then, 10μl CCK-8 reagent was added to each well at 0, 24, 48, 72 and 96 h, respectively. After that, all 96 well plates were cultured in 37°C incubator for 2 h, and the OD value at 450 nm was measured by spectrophotometer.

### Cell cycle was detected by flow cytometry

The cells in logarithmic phase were collected, and the single cell suspension was prepared with 0.25% trypsin, washed with PBS three times, and fixed overnight with 70% ethanol at 4°C. Propidium iodide (PI, Thermo Fisher, USA) was added according to the operation instructions, and then stored in the dark at 37°C for 30 min. flow cytometry (Becton- Dickinson, USA) was used for detection immediately. Cell cycle was analyzed by CELL Quest software.

### Apoptosis analysis by Annexin V/PI

The logarithmic phase cells were collected, and the single cell suspension was prepared with 0.25% trypsin, washed with PBS three times, and fixed without ethanol. Add Annexin V-fluorescein isothiocyanate (FITC)/propidium iodide (PI) (Thermo Fisher, USA) to the cell suspension according to the instructions. The apoptosis rate was detected by flow cytometry and analyzed by CELL Quest software.

### Cell migration was detected by transwell

Cell migration was detected by Transwell chambers (Costa, USA). The cell density was adjusted to 1.5 × 10^6^ cells/ml. 200 μl cell suspension was added into the upper chamber of Transwell, and 600 μl complete medium was added into the lower chamber. After 24 hours of culture, the cells were fixed with methanol and stained with crystal violet. Then, the cells were observed under the microscope (magnification, × 100).

### Quantitative real-time PCR (qRT-PCR)

Total RNA was isolated from tissues and cells by Trizol reagent (CA, USA). Then, the cDNA was synthesized with reverse transcription Kit (Applied Biosystems, Japan). Quantitative RT-PCR was performed using an ABI Prism 7500 instrument (Applied Biosystems) and SYBR Green PCR Master Mix kit (Takara, Japan). GAPDH was used as internal reference. The relative expression level of related genes was calculated by 2^−△△Ct^ method. All primers are provided by SANGON biotech (China) and are listed in Supplementary Table 1.

### Western blot assay

Firstly, the total protein was extracted by RIPA lysate (Thermo Fisher Scientific, USA) and analyzed by BCA protein quantitative kit (Thermo Fisher Scientific, USA). After that, the proteins were separated by 10% SDS-PAGE electrophoresis and transferred to PVDF membrane (Washington, New York). At room temperature, the membrane was sealed in 5% bovine serum albumin (BSA) for 1 h, and then incubated with primary antibody at 4°C overnight. The antibody was purchased from The antibodies were purchased from Cell Signaling Technology and were used at manufacturer-recommended dilutions. After that, the blot was incubated with HRP-conjugated secondary antibody (Santa Cruz, USA) at room temperature for 1 h. Finally, ECL kit (Thermo Fisher Scientific, USA) was used to observe the protein bands. GAPDH was used as internal reference.

### Dual-luciferase reporter assay

We synthesized the lncRNA GACAT1 and SFRP1 3′-UTR gene segments with either wide-type or mutant binds region and then subcloned the lncRNA GACAT1-WT, lncRNA GACAT1-MUT, SFRP1-WT, or SFRP1-MUT gene sequence into the psiCHECK-2 vector (Promega, USA). After that, these vectors were co-transfected into PECAPJ41 and HSC-4 cells with mimicNC or miR-149 mimics. After incubation for 48 h, luciferase activity was measured using the dual luciferase reporting analysis system (Promega USA).

### Statistical analysis

SPSS 20.0 software was used for statistical analysis. All data were expressed as mean ± standard deviation (SD). The data were analyzed by one-way ANOVA and student *t*-test. *P* < 0.05 was statistically significant.

## RESULTS

### GACAT1 is up-regulated and miR-149 is down-regulated in OSCC

**Figure 1:**
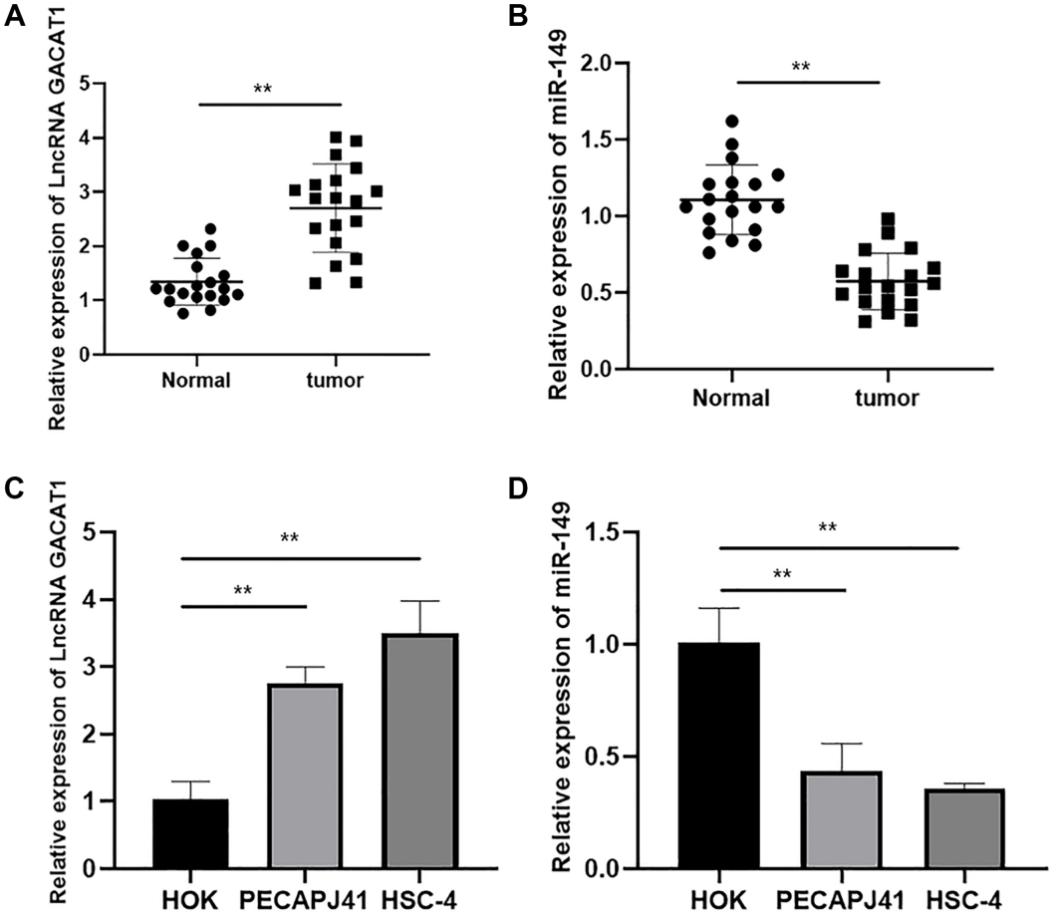
**Expression of lncRNA GACAT1 and miR-149 in cancer tissues and cell lines of patients with OSCC.** (**A**) Relative expressions of lncRNA GACAT1 in OSCC tissues; (**B**) Relative expressions of miR-149 in OSCC tissues; (**C**) Relative expressions of lncRNA GACAT1 in OSCC cell lines; (**D**) Relative expressions of miR-149 in OSCC cell lines. ^**^p < 0.01.

In order to investigate the role of GACAT1 and miR-149 in OSCC, we detected the expression of GACAT1 and miR-149 in OSCC tissues and adjacent normal tissues. As shown in Figure 1A and 1B, the expression of GACAT1 was significantly up-regulated and miR-149 was significantly down-regulated in OSCC tissues compared with adjacent normal tissues (*P* < 0.01). Then, we evaluated the expression of GACAT1 in OSCC cell lines (PECAPJ41 and HSC-4) and normal oral cells (HOK). As shown in Figure 1C–1D, compared with HOK cells, GACAT1 expression was significantly up-regulated and miR-149 expression was significantly down-regulated in PECAPJ41 and HSC-4 cells (*P* < 0.01).

### GACAT1 acted as a molecular sponge for miR-149

**Figure 2:**
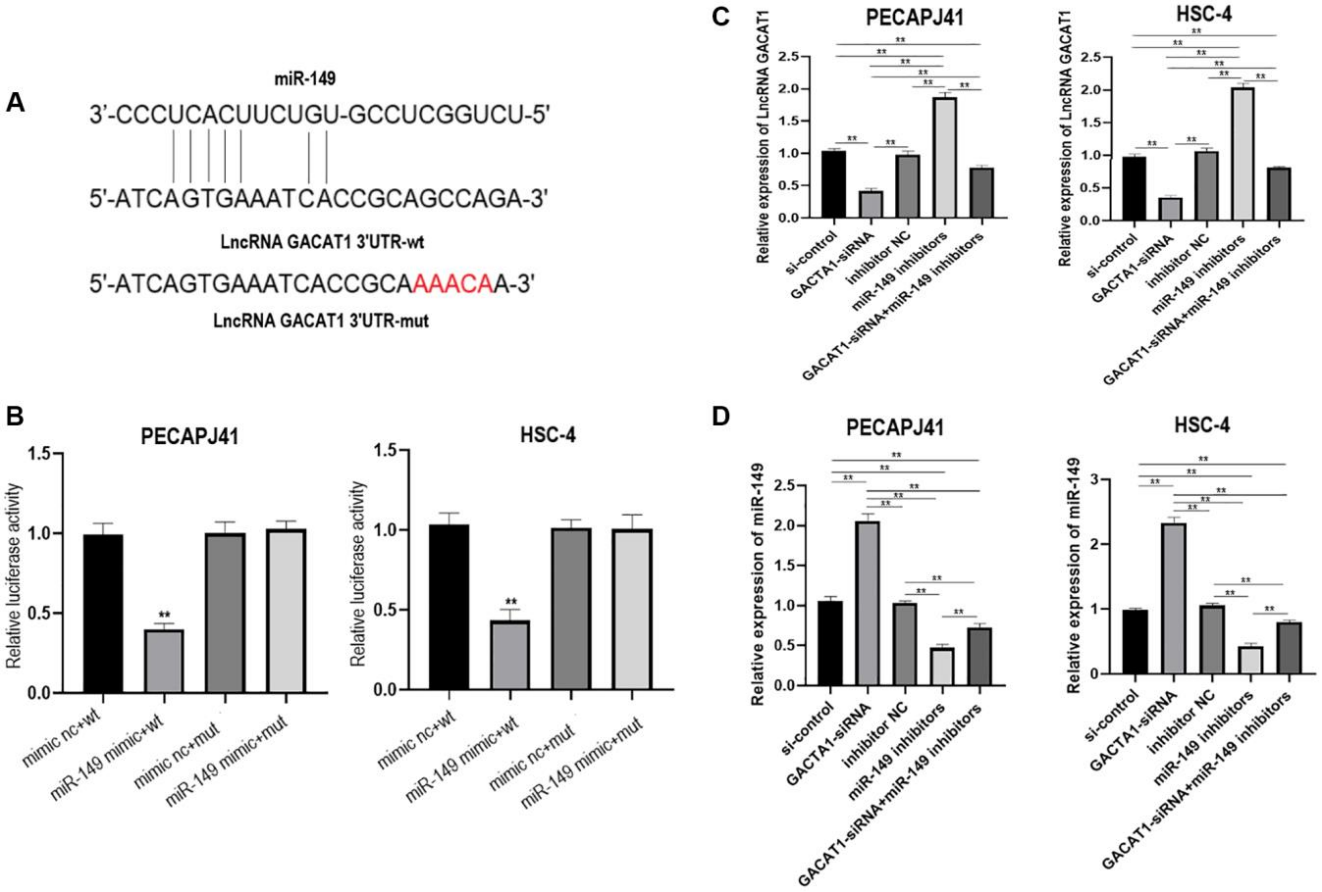
**lncRNA GACAT1 directly targeted miR-149 to regulate its expression.** (**A**) miRanda website predicted that lncRNA GACAT1 and miR-149 can be directly targeted to bind; (**B**) Transfection of lncRNA GACAT1-WT and miR-149 mimics inhibited luciferase activity in OSCC cells; (**C**) Effects of si-control, GACAT1-siRNA, inhibitor NC and miR-149 inhibitors on lncRNA GACAT1 expression in OSCC cells respectively or in combination; (**D**) Effects of si-control, GACAT1-siRNA, inhibitor NC and miR-149 inhibitors on miR-149 expression in OSCC cells respectively or in combination. ^**^p < 0.01.

Currently, lncRNAs are widely considered as “sponges” of miRNAs and may be involved in the process of OSCC. In this study, we explored whether GACAT1 could be used as a miRNA sponge, and predicted the potential binding sites of miR-149 in the GACAT1 sequence through the miRanda website (Figure 2A). The targeting relationship between GACAT1 and miR-149 was further verified by luciferase reporter analysis. The results showed that luciferase activity was decreased in OSCC cells when GACAT1-WT vector was co-transfected with miR-149 mimics (Figure 2B). In addition, qRT-PCR assay also confirmed the successful transfection of cells in each group (Figure 2C–2D).

### Effect of GACAT1 and miR-149 down-regulation on cell proliferation of OSCC

**Figure 3:**
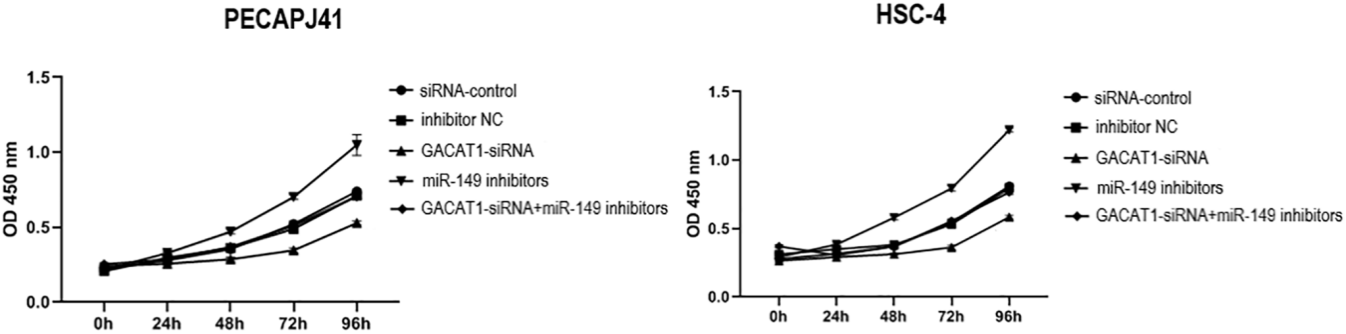
Effects of si-control, GACAT1-siRNA, inhibitor NC and miR-149 inhibitors respectively or in combination on the proliferation of OSCC cells.^**^p < 0.01.

We detected cell proliferation by CCK-8. Compared with si control group and inhibitor NC group, there was no significant change in cell proliferation in GACAT1 siRNA + miR-149 inhibitors group. Cell proliferation in GACAT1-siRNA group was significantly decreased, while that in miR-149 inhibitors group was significantly increased (*P* < 0.01) (Figure 3).

### Effect of GACAT1 and miR-149 down-regulation on cell cycle of OSCC

**Figure 4:**
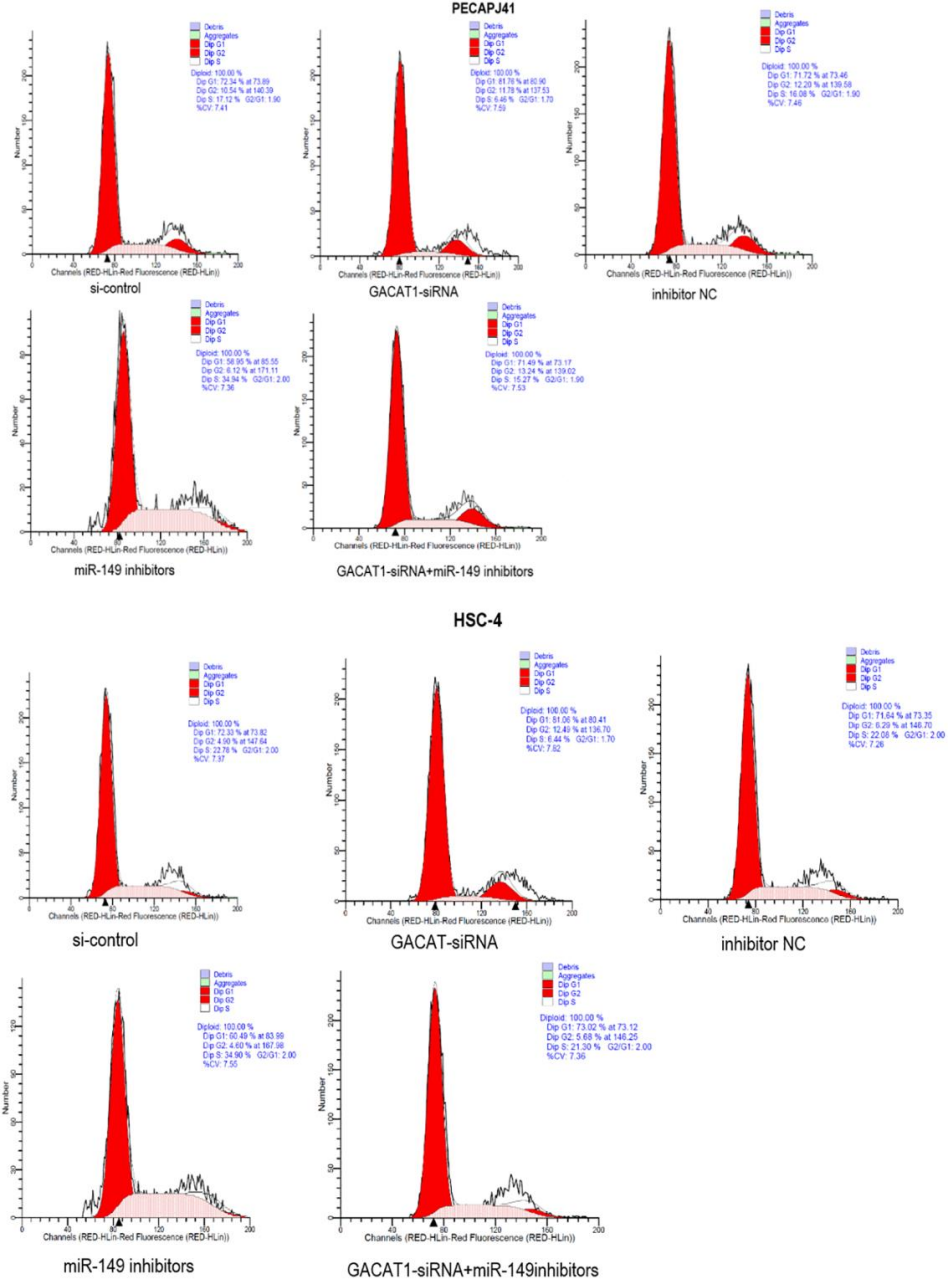
Effects of si-control, GACAT1-siRNA, inhibitor NC and miR-149 inhibitors respectively or in combination on the cell cycle of OSCC.^**^p < 0.01.

In this study, cell cycle results of PECAPJ41 and HSC-4 in each group were detected by flow cytometry. Compared with si-control group and inhibitor NC group, the cell cycle of GACAT1-siRNA + miR-149 inhibitors group was not significantly changed. The proportion of G0/G1 phase cells in the GACAT1-siRNA group increased, and the proportion of S phase cells decreased (*P* < 0.01). The proportion of G0/G1 phase cells decreased and the proportion of S phase cells increased in the miR-149 inhibitors group (*P* < 0.01). Compared with the GACAT1-siRNA group, the proportion of G0/G1 phase cells decreased in the miR-149 inhibitors group and the proportion of S-phase cells increased in the GACAT1-siRNA + miR-149 inhibitors group (*P* < 0.01). Compared with the miR-149 inhibitors group, the proportion of G0/G1 phase cells increased and the proportion of S phase cells decreased in the GACAT1-siRNA + miR-149 inhibitors group (*P* < 0.01) (Figure 4).

### Effect of GACAT1 and miR-149 down-regulation on apoptosis of OSCC

**Figure 5:**
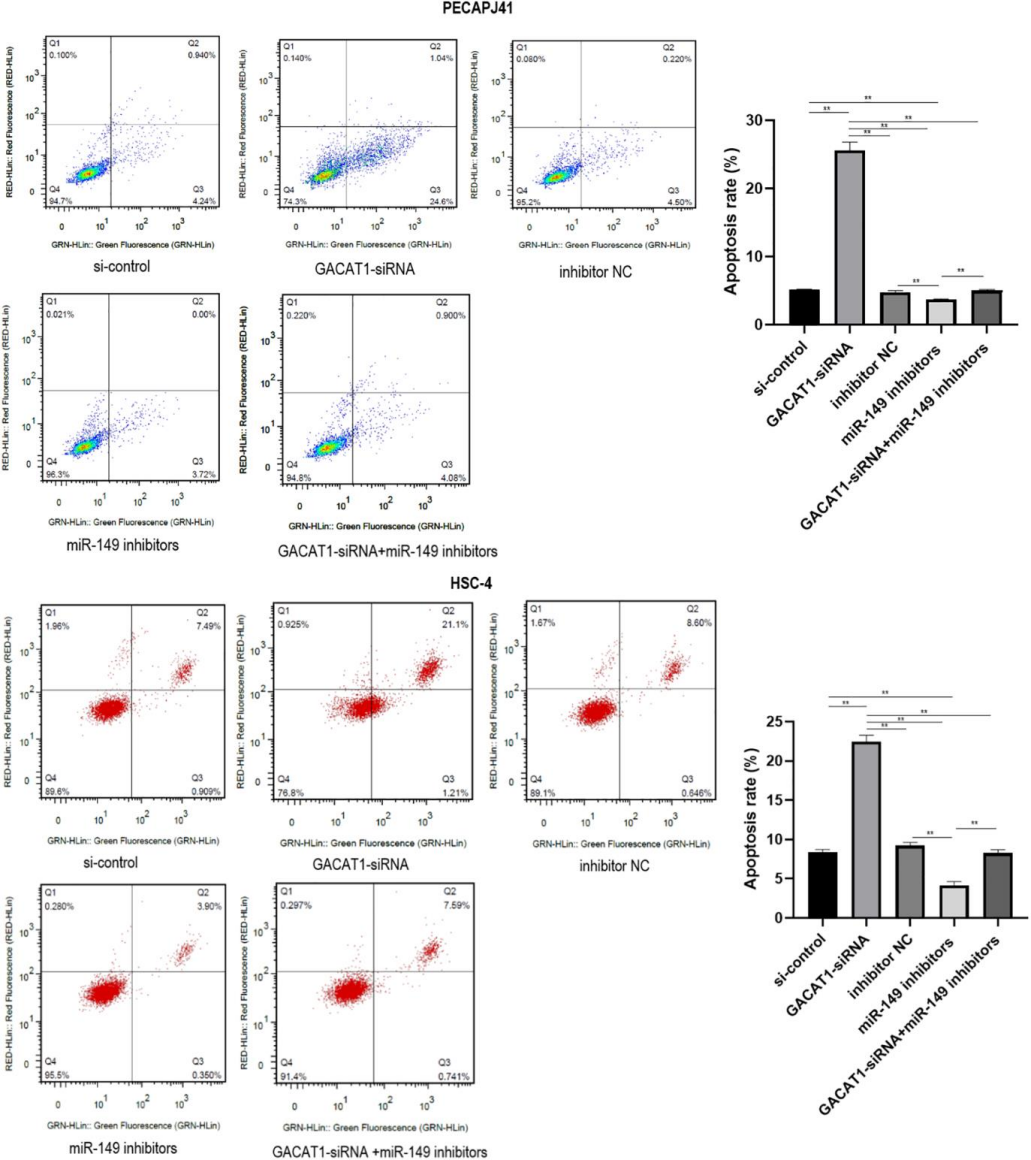
Effects of si-control, GACAT1-siRNA, inhibitor NC and miR-149 inhibitors respectively or in combination on apoptosis of OSCC cells.^**^p < 0.01.

Apoptosis results of PECAPJ41 and HSC-4 cells were shown in Figure 5. Compared with si-control group and inhibitor NC group, GACAT1-siRNA + miR-149 inhibitors group showed no significant change in cell apoptosis, while the cell apoptosis rate in the GACAT1-siRNA group was significantly increased (*P* < 0.01). The cell apoptosis rate in the miR-149 inhibitors group was significantly decreased (*P* < 0.01). The apoptosis rate of cells in the miR-149 inhibitors group and the GACAT1-siRNA + miR-149 inhibitors group was significantly lower than that in the GACAT1-siRNA group (*P* < 0.01). The apoptosis rate of cells in the GACAT1-siRNA + miR-149 inhibitors group was significantly increased compared to the miR-149 inhibitors group (*P* < 0.01).

### Effect of GACAT1 and miR-149 down-regulation on cell migration of OSCC

**Figure 6:**
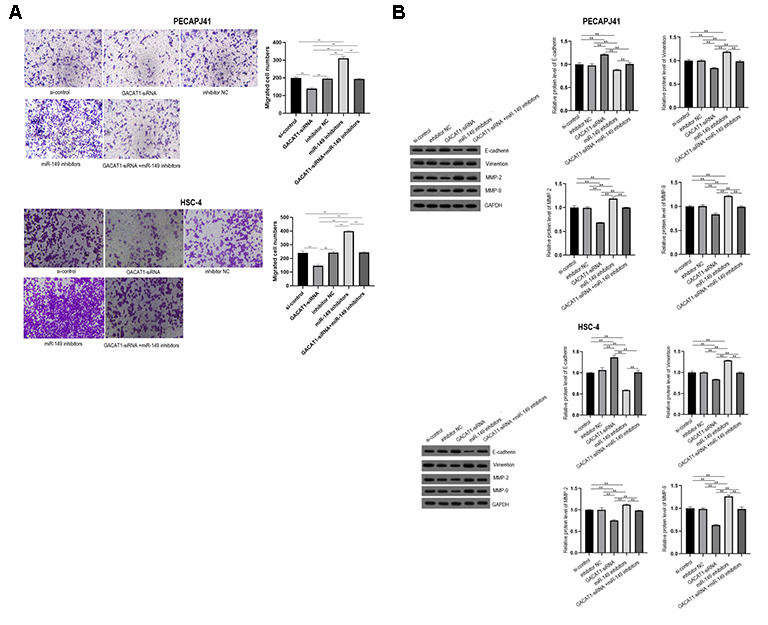
**Effects of si-control, GACAT1-siRNA, inhibitor NC and miR-149 inhibitors respectively or in combination on migration and related protein expression of OSCC cells.** (**A**) Transwell detected cell migration in each group; (**B**) The expression of E-Cadherin, Vimentin, MMP-2 and MMP-9 in each group were detected by Western blot assay. ^**^p < 0.01.

Transwell detected migration of PECAPJ41 and HSC-4 cells. GACAT1-siRNA + miR-149 inhibitors group showed no significant changes in cell migration compared with si-control group and inhibitor NC group. In the GACAT1-siRNA group, the adherent cell gap became larger, the number of cells decreased, and the ability of migration decreased (*P* < 0.01). In the miR-149 inhibitors group, the adherent cell gap became smaller, the cell number increased and the ability of migration was enhanced (*P* < 0.01). Compared with the GACAT1-siRNA group, the cell migration ability of the miR-149 inhibitors group and the GACAT1-siRNA + miR-149 inhibitors group was enhanced (*P* < 0.01). The cell migration ability of GACAT1-siRNA + miR-149 inhibitors group was decreased compared to the miR-149 inhibitors group (*P* < 0.01) (Figure 6A). In addition, we detected the expression of migration-related proteins in each group by Western blot. The results showed that, compared with the si-control group and inhibitor NC group, the expression trend of migration-related proteins in the GACAT1-siRNA + miR-149 inhibitors group was not significantly changed. Silencing GACAT1 could promote the expression of E-cadherin and inhibit the expression of Vimentin, MMP-2 and MMP-9 proteins. However, the expression trend of migration-related proteins in the miR-149 inhibitors group was opposite to that in the CAGAT1-siRNA group (Figure 6B).

### Effect of GACAT1 and miR-149 down-regulation on autophagy of OSCC cells

**Figure 7:**
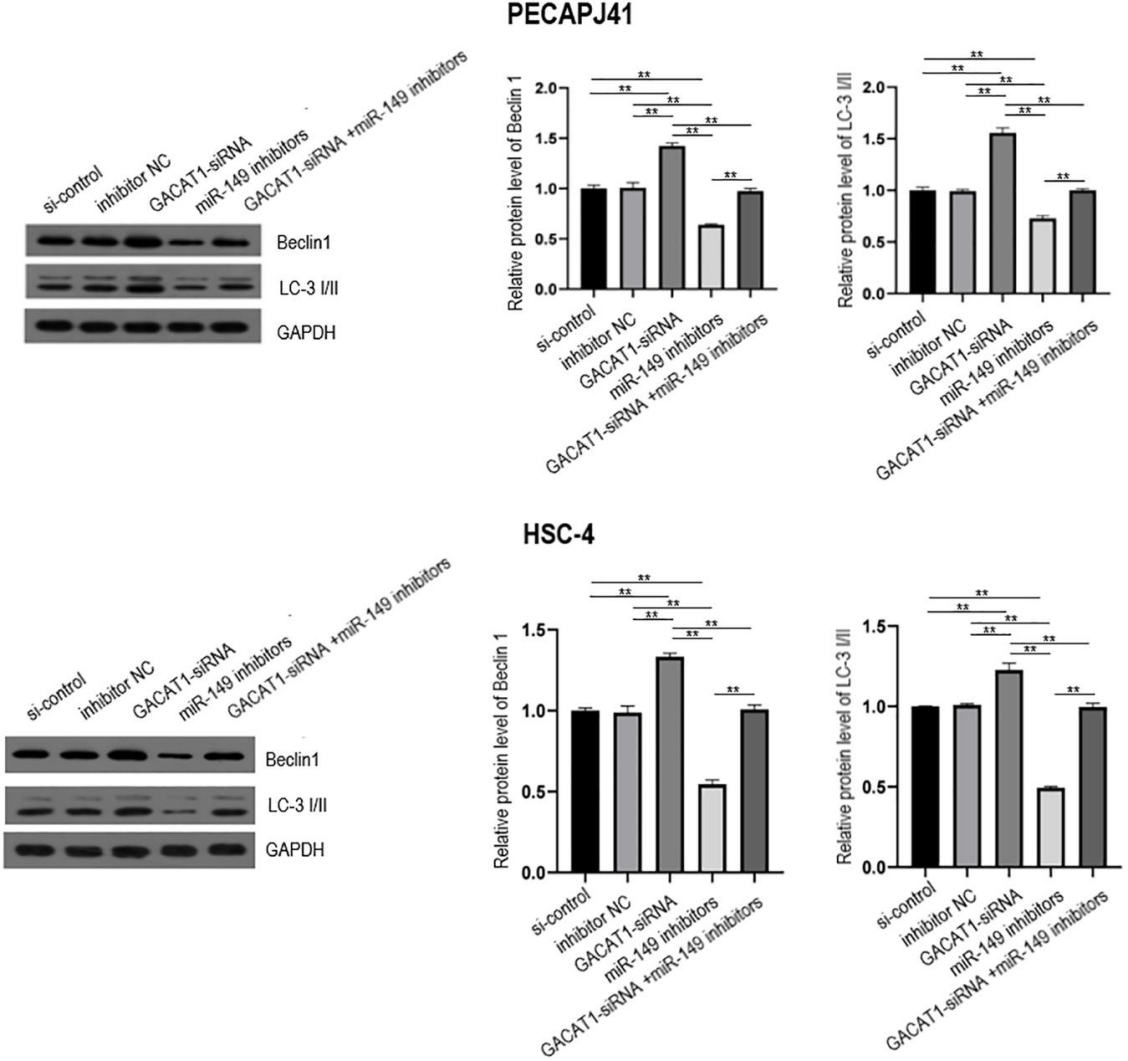
Western blot assay used to detect the effects of si-control, GACAT1 siRNA, inhibitor NC and miR-149 respectively or in combination on the expression of autophagy-related proteins Beclin1 and LC-3 I/II in OSCC cells.^**^p < 0.01.

In this study, the expression of autophagy-related proteins in each group was detected by Western blot. The results showed that, compared with the si-control group and inhibitor NC group, the expressions of Beclin1 and LC3 proteins in the GAVAT1-siRNA group were up-regulated (*P* < 0.01), the expression of miR-149 inhibitors group was down-regulated (*P* < 0.01), and there was no significant difference in the expression of GACAT1-siRNA + miR-149 inhibitors group. Beclin1 and LC3 protein expression was down-regulated in the miR-149 inhibitors group compared with the GACAT1-siRNA group (*P* < 0.01). Beclin1 and LC3 proteins were upregulated in the GACAT1-siRNA + miR-149 inhibitors group compared with the miR-149 inhibitors group (*P* < 0.01) (Figure 7).

## DISCUSSION

OSCC is a high-risk cancer, which seriously threatens the life and health of patients through local recurrence and metastasis, and its incidence is increasing year by year. Therefore, the potential molecular mechanisms that lead to OSCC have received more and more attention [13, 14]. In recent years, lncRNAs have been recognized as important factors in cancer progression. However, the potential functions and detailed regulatory mechanisms of most lncRNAs in human cancer remain unclear.

Gastric cancer-associated transcript 1 (GACAT1) gets its name from the fact that it was first found in gastric cancer [15]. Shi et al. found that the expression of GACAT1 is up-regulated in gastric cancer, and its overexpression can promote the proliferation and migration of gastric cancer cells [11]. Wang et al. reported that GACAT1 levels were significantly higher in breast cancer tissues than in paracancerous tissues [12]. However, GACAT1 has not been reported in OSCC. In this study, we demonstrated for the first time that lncRNA GACAT1 was up-regulated in 20 OSCC tissues and GC cells. In addition, we also found that GACAT1 knockout can significantly inhibit the proliferation and migration of OSCC cells, and promote apoptosis and autophagy. These results suggest that GACAT1 plays an oncogene role in OSCC, and inhibiting its expression is helpful to inhibit the occurrence and development of OSCC.

MiRNAs are evolutionarily conserved in structure and sequence and widely exist in organisms. They can regulate the expression of one or more genes after transcription and participate in cell proliferation, apoptosis, differentiation, individual growth and development and other life processes, which are closely related to the occurrence and development of tumors [16, 17]. Wang et al. found that the expression level of miR-21 in OSCC was significantly higher than that in the corresponding paracancerous tissues, and down-regulation of miR-21 expression could significantly inhibit the growth of OSCC cells and induce cell apoptosis [18]. It was also reported that the expression of miR-24 was up-regulated in OSCC tissues and patients’ plasma, which was related to the earlier clinical stage and shorter overall survival [19]. Silencing miR-24 could inhibit the proliferation of cancer cells [20]. miR-149 has been confirmed to play an important role in the occurrence and development of a variety of malignant tumors, and has the function of tumor suppressor gene. Feng et al. using the miRNA gene chip technology to filtered may participate in regulating the liver metastasis of microRNAs, the results showed that the expression of miR-149 abnormal amount is reduced, at the same time the research by qRT-PCR method in patients with liver cancer and normal plasma, found that miR-149 expression in liver cancer patients plasma and tissue levels significantly lower than normal plasma and corresponding normal tissue adjacent to carcinoma [21]. Schaefer et al. showed that the expression of miR-149 in prostate cancer tissues was significantly lower than that in corresponding adjacent normal tissues through miRNA microarray technology, and the results were verified by QRT PCR method to further confirm its authenticity [22]. However, compared with other types of malignant tumors, there are relatively few studies on miR-149 in OSCC, and its exact role in the occurrence and development of OSCC remains to be further explored. In this study, we found that the expression of miR-146 in OSCC tissues and cells was significantly lower than that in normal paracancerous tissues and normal oral cells. Inhibition of its expression can significantly promote the proliferation and migration of OSCC cells, and inhibit apoptosis and autophagy. These results demonstrate that miR-149 plays a role of tumor suppressor gene in OSCC.

Related studies have found that lncRNA can play the role of endogenous miRNA sponge, and then inhibit miRNA expression, indirectly affect the malignant biological behavior of tumor cells [23]. Wang et al. found that lncRNA LACAT1 can promote the malignant progression of OSCC by regulating the activity of miR-4301 [24]. Qu et al. demonstrated that lncRNA DANCR can promote the growth and metastasis of OSCC cells and inhibit the apotosis of OSCC cells by sponging miR-216a-5p [25]. In this study, we first predicted that there were regulatory sites between miR-149 and GACAT1 through miRanda website, and then verified by dual luciferase reporter gene detection experiment that GACAT1 targeted negative regulation of miR-149. Inhibition of miR-149 could reverse the effect of gacat1 silencing on apoptosis and autophagy of OSCC cells.

## CONCLUSIONS

In conclusion, GACAT1 can promote the proliferation and migration of OSCC cells, and inhibit apoptosis and autophagy, and the mechanism may be related to the targeted regulation of miR-149.This study provides a new target for the diagnosis, prevention and treatment of OSCC. However, there are still some deficiencies in this study. This study only confirmed the regulatory effect of GACAT1 on OSCC at the cellular level, but not at the clinical level, which limits the clinical significance of this study. Therefore, we need to further verify the clinical significance of this study in future clinical trials.

## Supplementary Materials

Supplementary Table 1
