# Supplementary material for: LncRNA GACAT1 targeting miRNA-149 regulates the molecular mechanism of proliferation, apoptosis and autophagy of oral squamous cell carcinoma cells
Source: Aging (Albany NY). 2021 Aug 30;13(16):20359–71. doi: 10.18632/aging.203416 (PMC8436912; doi:10.18632/aging.203416)
Supplement: Supplementary Table 1 [file aging-13-203416-s001.pdf]

SUPPLEMENTARY TABLE

Supplementary Table 1. Primer sequences.

Genes	Primers (5′ – 3′)
GACAT1	F: ACCGGAGGAAAATCCCTAGC<br>R: CCATAAAAGGGGCGGCTGT
miR-149	F: CATCCTTTCTGGCTCCGTGT<br>R: GCGTGATTCGTGCTCGTATATC
GAPDH	F: CCTCAAGATTGTCAGCAAT<br>R: CCATCCACAGTCTTCTGAGT
